# A Biohistorical Perspective of Typhoid and Antimicrobial Resistance

**DOI:** 10.1093/cid/ciz556

**Published:** 2019-10-15

**Authors:** Claas Kirchhelle, Zoe Anne Dyson, Gordon Dougan

**Affiliations:** 1 Wellcome Unit for the History of Medicine/Oxford Martin School, University of Oxford, Addenbrooke’s Hospital, United Kingdom; 2 Department of Medicine, University of Cambridge, Addenbrooke’s Hospital, United Kingdom

**Keywords:** typhoid, antimicrobial resistance, biosecurity, international health, vaccination

## Abstract

We combine methodology from history and genetics to reconstruct the biosocial history of antimicrobial resistance (AMR) in the bacterium *Salmonella enterica* serovar Typhi (*S*. Typhi). We show how evolutionary divergence in *S.* Typhi was driven by rising global antibiotic use and by the neglect of typhoid outside of high-income countries. Although high-income countries pioneered 1960s precautionary antibiotic regulations to prevent selection for multidrug resistance, new antibiotic classes, typhoid’s cultural status as a supposedly ancient disease of “undeveloped” countries, limited international funding, and narrow biosecurity agendas helped fragment effective global collective action for typhoid control. Antibiotic-intensive compensation for weak water and healthcare systems subsequently fueled AMR selection in low- and middle-income countries but often remained invisible due to lacking surveillance capabilities. The recent rise of extensively drug-resistant typhoid bears the biosocial footprint of more than half a century of antibiotic-intensive international neglect.

The past 2 decades have seen a technological revolution provide deeper insight into human and infectious disease history. The rapid evolution of DNA sampling and sequencing has yielded a clearer understanding of the evolutionary trajectory of pathogens ranging from *Yersinia pestis* (plague) to *Vibrio cholerae* (cholera) [1–4]. The interpretation of data yielded by this revolution has not been uncontested. Historians, archaeologists, microbiologists, and other disciplines have debated the impact of selective sampling, phylogenetic theories, rapid turnover, small sample sizes, “historical cherry-picking” [5], and retrospective diagnoses [6–10]. However, the debate is also forcing biologists to critically engage historical sources and historians to familiarize themselves with a new world of sequences and phylogenies [1, 11–14].

Co-written by a historian, a microbiologist, and a bioinformatician, this article uses the example of antimicrobial resistance (AMR) in the bacterium *Salmonella enterica* serovar Typhi (*S*. Typhi), the cause of human typhoid, to explore an interdisciplinary sociomaterial approach to infectious disease history [15]. An integrated approach is necessary; knowing the complete details of a bacteria’s genome tells us relatively little about the historical impact of a disease but to neglect microbial materiality is to ignore important data [16]. Building on work by the anthropologist Hannah Landecker [17], our fusion of methodologies reveals a story of evolutionary diversification in *S.* Typhi driven by antimicrobial selection pressure, fragmented international policy, and narrow biosecurity agendas. We suggest that from the 1960s onward, growing concerns about resistant typhoid did not result in coordinated global collective action to curb drivers of typhoid outside of resource-rich settings. Despite receiving high-income country (HIC) support during acute outbreaks and after natural disasters, the lack of effective surveillance, preventative sanitary infrastructures, and vaccine programs in many low- and middle-income countries (LMICs) likely encouraged reliance on antimicrobials and resulting AMR selection. The recent rise of extensively drug-resistant (XDR) typhoid is a result of these fragmentary global control efforts.

## BIOGRAPHY OF A DISEASE

The invention of scalable DNA sequencing by Fred Sanger and colleagues in the 1980s stimulated the use of forensic DNA analysis for both humans and bacteria. Over the past decade, Sanger sequencing and subsequent technologies have yielded unprecedented insight into the biological history of typhoid. As bacteria replicate, they accumulate mutations, for example in the form of single-base pair nucleotide polymorphisms (SNPs), that provide a traceable footprint of their ancestry. The family histories or phylogeny of clades of bacteria can be established by SNP typing or sequencing of historical collections [18–21]. During the 2000s, next-generation sequencing techniques took forensic DNA analysis to a new level, allowing the rapid definition of phylogeny based upon the complete genome sequences of hundreds or thousands of bacteria [22, 23]. These approaches can also be used to define the emergence of AMR in bacteria and trace the origins of outbreaks over time and geography [24–27].

**Figure 1.**
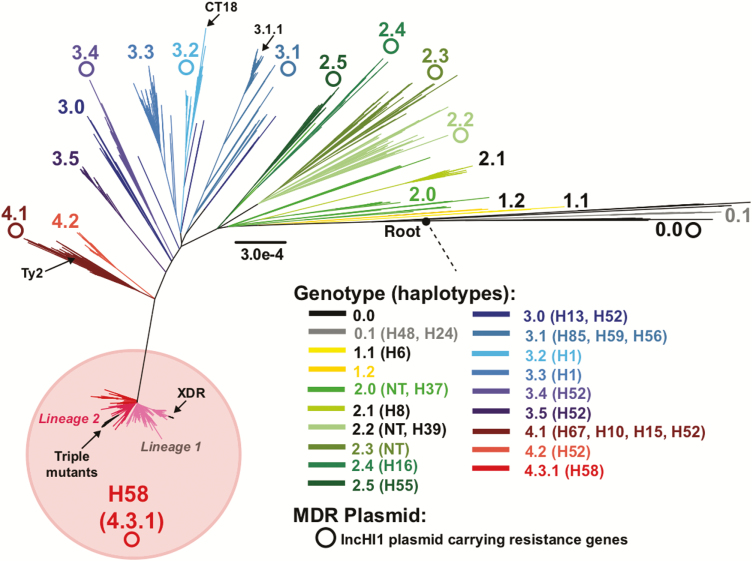
Maximum likelihood phylogeny based on single-nucleotide polymorphism data from a global collection of 3748 *Salmonella* Typhi strains. Branches are colored by genotype/lineage (clade level) according to the inset legend and labels. These span out from the ancestral origin. Haplotypes are indicated in the inset legend, with NT indicating nontypeable strains under the Roumagnac scheme [18]. Arrows indicate fluoroquinolone-resistant triple mutant clade, Pakistan extensively drug-resistant (XDR) clade, West African 3.1.1 multidrug-resistant (MDR) clade, as well as CT18 (MDR reference strain originally in Vietnam in 1993) and strain Ty2 (classical old Typhi from 1912). Pink shading indicates H58 (4.3.1) strains that have undergone clonal expansion. Open circles indicate that a strain harboring an IncHI1 MDR plasmid carrying antimicrobial resistance genes has been observed in a member or members of the clade (colored by lineage according to the inset legend). The closed circle indicates the ancestral root, and the dashed line represents the *Salmonella* lineage. Branch lengths are indicative of the estimated substitution rate per variable site as per the inset scale bar. Note expansion of H58.

For individual diseases, sequence-based analysis can provide estimates of the age of the pathogen and insight into its origin. For human typhoid, we can estimate that the disease is no more than a few thousand years old, although this still has to be properly calculated using the large datasets now available. Phylogenetic analysis shows that all current *S*. Typhi originated from one bacterium that moved into the human population, perhaps from a primate, and lost the ability to infect and transmit within other animal populations. Figure 1 shows a simplified family tree of *S*. Typhi based on SNPs detected in previously sequenced strains (Supplementary Table 1 and Supplementary Methods) [21, 28–33]. This tree can be used as a template to genetically type any *S*. Typhi (see Figure 1 legend) and it clearly illustrates the ancestral origin and selected antibiotic-resistant lineages. For convenience, related bacteria are clustered into genotypes (also known as haplotypes) that can represent particular lineages descended from a common ancestor within the tree.

## A GOLDEN AGE OF CONTROL

For the longest part of its natural history, *S.* Typhi was both optically and culturally invisible. Pathologists only began to differentiate between typhoid and other fevers from the mid-19th century onward and the organism *S.* Typhi was isolated and linked to typhoid fever between 1880 and 1884. New knowledge of typhoid informed targeted control measures. Over the next decades, sanitary interventions, new vaccines, gall bladder removals (cholecystectomy), and carrier identification significantly reduced disease incidence in HICs (Vanderslott et al in this supplement [paper 2]). However, it was only from around 1940 onward that researchers considered systematic typhoid eradication schemes. Two then-new technologies inspired eradication plans: epidemiologically driven bacteriophage typing (using bacteria infecting viruses to identify individual bacteria strains) and effective chemotherapy in the form of antibiotics [34].

In the case of bacteriophage typing, the reduced overall typhoid incidence and centralized bacteriological capabilities allowed researchers to identify and control outbreaks and then systematically seek and register asymptomatic (healthy) carriers. The aim was to dry out endemic reservoirs by educating—and occasionally isolating—carriers. After 1945, global phage typing surveys facilitated outbreak monitoring and the identification of unknown carriers among civilian populations, food handlers, travelers, and military personnel returning from abroad [35]. Parallel to the typing and mapping of typhoid strains, clinicians experimented with new mass-produced antibiotics to treat outbreaks and stop carrier-mediated shedding. Initial trials with sulphonamides, penicillin, and tetracyclines proved disappointing [36–39]. However, in 1948, US Army researchers in Malaya reported that Parke-Davis’ Chloromycetin (chloramphenicol) cured patients during typhoid outbreaks [40]. In 1961, ampicillin emerged as a second-line treatment, which was also partially effective in curing carriers [41].

## TYPHOID AND ANTIBIOTIC STEWARDSHIP

Hopes of typhoid eradication were quickly threatened by the emergence of antibiotic-resistant *S*. Typhi. Following reports describing typhoid resistance against individual antibiotics during the 1950s, a growing number of *S.* Typhi isolates from around the world proved resistant to multiple antibiotics (MDR) from the 1960s onward.

In HICs, typhoid’s cultural status as a newly defeated scourge meant that fears of reemerging untreatable strains played a significant role in structuring the first wave of environmental antibiotic stewardship regulations. During the 1950s, medical concerns about resistance against sulphonamides and other antibiotics in organisms like *S. aureus* had already led to campaigns for rational antibiotic use [42, 43]. In the case of *S.* Typhi, rising chloramphenicol resistance in individual patients was reported within 2 years of the first trials in 1950 [44]. However, it was only between 1958 and 1965 that the Japanese discovery of AMR transfer in *Shigella* and British data on agricultural resistance transfer led to popular concerns about a return to the “pre-antibiotic Middle Ages” [45]. Public health experts warned that AMR was “infectious” and could move between bacterial species by mechanisms such as plasmid-mediated conjugation.

During the 1960s and 1970s, the European campaign for precautionary antibiotic restrictions was led by Ephraim Saul (E. S.) Anderson, director of Britain’s Public Health Laboratory Service enteric reference laboratory. Having only recently dealt with a 400-case typhoid outbreak in Aberdeen in 1964 [46], Anderson used evidence of transferable multiple resistance in human and animal *Salmonella* Typhimurium isolates to highlight the dangers of mass-environmental AMR selection in 1965 [47–52]. The specter of transferable ampicillin and chloramphenicol resistance leading to untreatable typhoid encouraged Anderson to advocate precautionary bans of growth promoter feeds containing medically relevant antibiotics in livestock production. Targeted growth promoter bans were subsequently approved across Western Europe (1969 & 1970) but failed to gain congressional approval in the United States (1972 & 1977) [53].

## TRANSFORMING INTO A FOREIGN DISEASE

The enactment of precautionary antibiotic restrictions in Europe marked an early high-point of popular awareness for AMR in typhoid. However, over the next decades, *S.* Typhi’s effective disappearance from many HICs inadvertently divided international perceptions of the disease. While increasingly resistant typhoid variants remained endemic in many LMICs, public commentators in HICs reemphasized historic connotations of typhoid as an “ancient” disease associated with allegedly “primitive” conditions in less developed foreign countries whose main threat to HICs consisted in carriage by travelers and immigrants [54–57]. In line with an increasing shift of international health policies away from sociomedical (horizontal) to allegedly more cost-effective biotechnological interventions (vertical) [58, 59], typhoid was increasingly framed in HICs not as a collective global challenge of LMIC system strengthening but as a security threat that had to be stopped from crossing national borders.

Worryingly, shifting HIC typhoid perceptions occurred right at the moment when 1960s scenarios of transferable multiple resistance in typhoid were becoming a reality in LMICs. During the 1970s, explosive outbreaks of *S.* Typhi with plasmid-mediated resistance against multiple antibiotics including chloramphenicol occurred in Central America, India, and Vietnam. Emerging in endemic areas with widespread antibiotic use and often poor health and water infrastructures [60], the outbreaks resembled those predicted by E. S. Anderson. Fortunately, early plasmids had not acquired ampicillin resistance, although this did eventually appear [61]. HIC responses to these outbreaks frequently prioritized national biosecurity measures over collective preventive action. Following on the heels of a multiresistant *Shigella dysenteriae* outbreak, the 1972–1974 Mexican typhoid outbreak with >10 000 confirmed cases was influential in reinforcing biosecurity-focused HIC typhoid policies [62, 63]. In the United States, the Mexican outbreak occurred parallel to a significant epidemiological transition whereby nearly half of new typhoid cases were being imported from abroad. 1970s publications by US public health officials not only highlighted the contemporary doubling of cases in Los Angeles but also longer-term correlations of typhoid incidence with alleged “Hispanic” surnames, traveling and hygiene habits, as well as with rising licit and illicit border traffic and immigration [60, 64, 65]. Although they supported Mexican colleagues to phage type and analyze outbreaks [63, 66, 67], a significant part of US officials’ subsequent work focused on upgrading domestic epidemiological surveillance and promoting hygiene and vaccination among travelers to stop foreign strains from entering the United States [68–72]. European authorities also closely monitored travelers while media reports chided Latin Americans and former colonies for failing to implement antibiotic restrictions and growth promoter bans [73–75].

The increasing prioritization of border protection did not mean that international aid for typhoid control stopped: During the 1970s and 1980s, states on both sides of the Iron Curtain provided economic, infrastructural, and expert support to allies while charities like Oxfam administered free typhoid vaccines in the wake of natural disasters like the 1973 Pakistan floods. However, bilateral and charitable interventions were often too short-term, underresourced, and statist to alleviate the sanitary and health gaps contributing to typhoid incidence and antibiotic overuse in endemic countries without already robust health bureaucracies [59, 76–80]. Underresourcing was mirrored at the international level. Between 1972 and 1978, calls by the United Nations (UN) Stockholm Conference (1972), the UN Mar Del Plata Conference (1977), and the International Conference on Primary Health Care at Alma-Atta (1978) increased foreign aid, credit provision, and investment in water and health systems. However, despite the UN’s designation of an International Decade of Water between 1981 and 1990, these “horizontal” initiatives did not generate sufficient funds to keep pace with global population growth and urbanization [58, 81–83].

Buoyed by the success of global smallpox eradication (1980) and a rising HIC focus on cost-effective interventions during a decade of economic instability, the 1980s instead experienced renewed international emphasis on vertical biotechnological control measures such as vaccines and oral rehydration therapy to combat enteric disease [58, 59]. In the case of typhoid, inactivated bacterial vaccines (phenol, heat, or acetone killed) had traditionally been used to protect travelers and military personnel [84]. Aside from outbreaks, wider public health use of these reactogenic vaccines had been limited. During the 1972–1973 Mexican outbreak, >5 million doses of inactivated vaccines were administered alongside other hygiene measures and ampicillin treatment. However, it remained unclear in how far mass vaccination had curbed the outbreak and whether ongoing vaccination could further shrink endemic disease reservoirs [67, 85, 86]. Around 1980, new hopes for vaccine-based typhoid control centered on an oral vaccine based on a live attenuated *S.* Typhi (Ty21a) strain. First described in 1975, multiple genes, including those involved in the expression of the Vi capsular polysaccharide, had been chemically mutated to render Ty21a harmless. The commercially developed vaccine was tested in Egypt (1978), Chile (1982, 1983, 1984 & 1986), and Indonesia (1986). Trials were closely monitored by the World Health Organization (WHO) and US experts and helped control a typhoid mass outbreak in Santiago, Chile [87–90]. In addition to Ty21a, new parenteral vaccines using the Vi antigen to induce immunity were successfully tested in South Africa and Nepal. While the relative value of mass vaccination as opposed to sanitary interventions for disease control in endemic settings remained subject to debate until the 1990s [91], the ease of use, efficacy, and low reactogenicity of the new typhoid vaccines significantly increased human protection [92, 93].

Alongside vaccine development, new effective antibiotics (trimethoprim-sulfamethoxazole, 1974) further contributed to the feeling that the 1970s burst of MDR typhoid variants posed no serious hazard to global health outside of endemic areas. As late as 1984, participants at an international workshop on typhoid—one of 4 global surveys of typhoid since 1955—noted that better diagnostics, new vaccines, and allegedly unstable *S.* Typhi R-factors “engendered a sense of optimism among participants for improved, worldwide control of typhoid fever” [94].

However, in the absence of effective measures reducing the hazards of unclean water, inadequate healthcare, and resulting antibiotic overuse in many parts of the world, this optimism proved premature. Four years after the 1984 workshop, *S.* Typhi strains isolated during a typhoid outbreak among 230 people in Kashmir proved resistant to all 3 first-line drugs. MDR was plasmid-mediated and transferable to *Escherichia coli.* Strains with transferable resistance were also isolated in Shanghai. In the early 1990s, further MDR outbreaks were reported in Pakistan and the Mekong Delta [95].

## LOSING CONTROL

In addition to typhoid’s relatively low priority in relation to other more lethal or fast-burning diseases [96, 97] and the rising international cost-benefit focus on vaccines and biosecurity, poor surveillance capabilities in many endemic areas help explain why many experts initially underestimated seemingly sporadic MDR outbreaks. Surveillance gaps changed little over the next 3 decades. In the absence of funds, LMIC laboratories capable of diagnosing typhoid by culture and testing isolates for AMR were relatively rare and typhoid and AMR burdens remained invisible.

One exception was Vietnam where the Wellcome Trust had established a laboratory in Ho Chi Minh City in 1991. By the mid-1990s, Wellcome and Vietnamese researchers were reporting the isolation and characterization of MDR *S*. Typhi. Importantly, molecular epidemiological techniques, including pulsed-field gel electrophoresis [98], were used to subtype the *S*. Typhi and it became clear that a particular pulsed-field type associated with MDR had dominated the typhoid epidemiology since around 1990. This new type appeared to be replacing other *S*. Typhi subtypes.

A clearer epidemiological and microbiological picture emerged following the development of new DNA sequence–based typing schemes in the late 1990s. When SNP typing was first applied to a small global collection of approximately 80 *S*. Typhi (including MDR) isolates, a group of isolates fell into a genetically restricted clade known as haplotype H58 [18]. Haplotype H58 was deemed to be undergoing a rapid population expansion that was confirmed through whole genome–based DNA sequence analysis of >2000 *S*. Typhi samples covering approximately 60 countries [99]. Approximately 50% of recent *S*. Typhi isolates were H58 (genotype 4.3.1, see Figure 1) and typing indicated that H58 was spreading into regions, including much of East Africa, where typhoid had previously been relatively rare or underreported.

Similar to the reemergence of other “ancient” diseases like tuberculosis [100–102], genetic change and geographic expansion in *S.* Typhi subpopulations were being driven by social factors like poverty, international neglect, regional instability following the collapse of the Soviet Union, and antibiotic intensive compensation for lacking healthcare [91, 103, 104]. Between the 1980s and 1990s, rapidly increasing global antibiotic use made the efficacy of newer first- and second-generation fluoroquinolones (norfloxacin, 1978; ofloxacin, 1980/1985; ciprofloxacin, 1987), macrolides (azithromycin, 1980/1986), and third-generation cephalosporins (ceftriaxone, 1984) against typhoid short lived [105–107]. After 1993 reports of sporadic quinolone resistance, >90% of strains from a 1996–1998 outbreak affecting 24 000 people in war-torn Tajikistan were MDR and 82% resistant to ciprofloxacin. Despite rising international AMR warnings and 1994 WHO proposals to use vaccines to control resistant typhoid [108, 109], ongoing global overuse of third-generation cephalosporins and fourth-generation fluoroquinolones (gatifloxacin, 1999) led to further AMR selection [29, 31, 110, 111]. In the case of the cephalosporins, sporadic ceftriaxone resistance was reported during the early 2000s and Pakistan’s current outbreak is proving resistant to all first-line drugs, the quinolones, and ceftriaxone. Interestingly, XDR *S*. Typhi isolates from this outbreak were H58s that had been infected by a promiscuous multidrug resistance plasmid normally found in *E. coli* and other enteric pathogens—thus confirming Anderson’s 1960s warnings about environmental AMR selection. For many low-income victims, azithromycin is now the last effective and affordable antibiotic. Azithromycin-resistant strains already exist [61, 112].

## CONCLUSIONS

XDR H58 is the most recent and worrying chapter of typhoid’s biosocial history. However, its rise continues to be overshadowed by other, more lethal or fast-burning diseases. According to Google’s NGram viewer, popular awareness for “typhoid” is far lower than during the mid-20th century. In the absence of effective global surveillance and media coverage, the high-income publics continue to view typhoid as a “foreign” disease of the past [103].

This perception has contributed much to typhoid’s current biology. Between the 1940s and 1970s, effective sanitation, surveillance, vaccination, and a golden age of effective chemotherapy nearly eliminated typhoid incidence in HICs. The frequently invoked success story of Western typhoid control also enabled European public health campaigners to use the specter of reemerging resistant typhoid to push for precautionary bans of agricultural antibiotics. The irony is that these precautionary bans were implemented right at the time when poor sanitary and healthcare infrastructures as well as increasing nontargeted antibiotic use in the Global South fostered a drug-resistant resurgence of the disease. This resurgence did not trigger a systematic global collective response. Between the 1970s and 1990s, wealthier typhoid-free countries focused on protecting travelers and preventing *S.* Typhi from crossing borders. Meanwhile, international underfinancing and a growing focus on vertical technological interventions stalled the spread of sanitary and health infrastructures that had originally helped curb typhoid in HICs and whose absence continues to be a significant driver of antibiotic overuse and AMR in LMICs [113]. International neglect and antibiotic overuse imprinted on *S.* Typhi’s biology: The ongoing XDR-H58 outbreak bears the biohistorical footprint of half a century of narrow priorities and fragmented international antibiotic stewardship.

## Supplementary Data

Supplementary materials are available at *Clinical Infectious Diseases* online. Consisting of data provided by the authors to benefit the reader, the posted materials are not copyedited and are the sole responsibility of the authors, so questions or comments should be addressed to the corresponding author.

ciz556_suppl_Supplemental-Table-1

ciz556_suppl_Supplemental-Methods

## Notes

***Acknowledgments.*** The authors acknowledge help from Professor Andrew Pollard, Professor Stephen Baker, and Dr Samantha Vanderslott in editing this manuscript.

***Financial support.*** G. D. was supported by the Wellcome Trust (Strategic Typhoid alliance across Africa and Asia [STRATAA]) and the Bill & Melinda Gates Foundation (TyVAC). Z. A. D. was supported by a grant funded by the Wellcome Trust (STRATAA).

C. K. reports grants from the Higher Education Funding Council for England Global Challenges Research Fund and the New Venture Fund to support work on and publication of this supplement.

***Supplement sponsorship.*** This supplement has been funded by the New Venture Fund.

***Potential conflicts of interest.*** All authors: No reported conflicts of interest. All authors have submitted the ICMJE Form for Disclosure of Potential Conflicts of Interest.
