# Supplementary material for: A Biohistorical Perspective of Typhoid and Antimicrobial Resistance
Source: Clin Infect Dis. 2019 Oct 15;69(Suppl 5):S388–94. doi: 10.1093/cid/ciz556 (PMC6792120; doi:10.1093/cid/ciz556)
Supplement: ciz556_suppl_Supplemental-Methods [file ciz556_suppl_supplemental-methods.docx]

**Supplementary methods**

**Read alignment and Single Nucleotide Polymorphism (SNP) analysis**

*S.* Typhi Illumina reads from 3,747 isolates sequenced in previous studies [1-8] and listed in **Supplementary table 1** were mapped to the CT18 (accession no. AL513382) reference chromosome sequence [9] using the RedDog mapping pipeline (v1beta.10.3; <https://github.com/katholt/reddog>). Briefly, RedDog uses Bowtie (v2.2.9) [10] to map reads to the reference genome, and SAMtools (v1.3.1) [11] to identify SNPs that have a phred quality score >30, and to filter out those SNPs supported by <5, or with 2.5x the average read depth that represent putative repeated sequences, or those that have ambiguous base calls. For each SNP that passes these criteria in any one isolate, consensus base calls for the SNP locus were extracted from all genomes mapped, with those having phred quality scores <20 being treated as unknown alleles and represented with a gap character. These SNPs were then used to assign isolates to previously defined genotypes according to an extended *S.* Typhi genotyping framework using the GenoTyphi software tool, available at <https://github.com/katholt/genotyphi> [3, 12].

Chromosomal SNPs with confident homozygous calls (phred score >20) in >95% of the genomes mapped (representing a ‘soft’ core genome) were concatenated to form an alignment of alleles at 38,844 variant sites. SNPs called in prophage regions and repetitive sequences (354 kb; ~7.4% of bases in the CT18 reference chromosome, as defined previously [1, 8] or in recombinant regions as detected by Gubbins (v2.3.2) [13] were excluded resulting in a final alignment of 31,771 chromosomal SNPs out of a total alignment length of 4,462,203 bp for 3,748 *S.* Typhi genomes. SNP alleles from *S.* Paratyphi A AKU1_12601 (accession no: FM2000053) were included in the alignment for outgroup rooting of the phylogenetic tree.

**Phylogenomic analysis**

A maximum likelihood (ML) phylogenetic trees was inferred from the aforementioned chromosomal SNP alignments using RAxML (v8.2.8) [14]. A generalized time-reversible model and a Gamma distribution was used to model site-specific rate variation (GTR+ Γ substitution model; GTRGAMMA in RAxML) with 100 bootstrap pseudoreplicates used to assess branch support for the ML phylogeny. The resulting phylogeny was visualised using FigTree [15].

**Detection of Plasmids and Antimicrobial Resistance genes**

The mapping based allele typer SRST2 [16] was used in conjunction with the ARG-ANNOT [17] and PlasmidFinder [18] databases to detect acquired AMR genes and plasmid replicons in raw *S.* Typhi read data.

**References**

1. Wong VK, et al. An extended genotyping framework for Salmonella enterica serovar Typhi, the cause of human typhoid. Nature Communications **2016**; 7: 12827.

2. Wong VK, Holt KE, Okoro C, et al. Molecular surveillance identifies multiple transmissions of typhoid in West Africa. PLoS neglected tropical diseases **2016**; 10(9): e0004781.

3. Britto CD, Dyson ZA, Duchene S, et al. Laboratory and molecular surveillance of paediatric typhoidal Salmonella in Nepal: Antimicrobial resistance and implications for vaccine policy. PLoS neglected tropical diseases **2018**; 12(4): e0006408.

4. Klemm EJ, Shakoor S, Page AJ, et al. Emergence of an extensively drug-resistant Salmonella enterica serovar Typhi clone harboring a promiscuous plasmid encoding resistance to fluoroquinolones and third-generation cephalosporins. MBio **2018**; 9(1): e00105-18.

5. Park SE, Pham DT, Boinett C, et al. The phylogeography and incidence of multi-drug resistant typhoid fever in sub-Saharan Africa. Nature Communications **2018**; 9(1): 5094.

6. Tanmoy AM, Westeel E, De Bruyne K, et al. Salmonella enterica Serovar Typhi in Bangladesh: Exploration of Genomic Diversity and Antimicrobial Resistance. mBio **2018**; 9(6): e02112-18.

7. Thanh DP, al. e. A novel ciprofloxacin-resistant subclade of H58 Salmonella Typhi is associated with fluoroquinolone treatment failure. Elife **2016**; 5: e14003.

8. Ingle DJ, Nair S, Hartman H, et al. Informal genomic surveillance of regional distribution of Salmonella Typhi genotypes and antimicrobial resistance via returning travellers. bioRxiv **2019**: 510461.

9. Parkhill J, Dougan G, James KD, et al. Complete genome sequence of a multiple drug resistant Salmonella enterica serovar Typhi CT18. Nature **2001**; 413(6858).

10. Langmead B, Salzberg SL. Fast gapped-read alignment with Bowtie 2. Nature methods **2012**; 9(4): 357.

11. Li H, Handsaker B, Wysoker A, et al. The sequence alignment/map format and SAMtools. Bioinformatics **2009**; 25(16): 2078-9.

12. Wong V, Baker S, Connor T, et al. International Typhoid Consortium. An extended genotyping framework for Salmonella enterica serovar Typhi, the cause of human typhoid. Nature Communications **2016**; 7: 12827.

13. Croucher NJ, Page AJ, Connor TR, et al. Rapid phylogenetic analysis of large samples of recombinant bacterial whole genome sequences using Gubbins. Nucleic acids research **2014**; 43(3): e15-e.

14. Stamatakis A. RAxML-VI-HPC: maximum likelihood-based phylogenetic analyses with thousands of taxa and mixed models. Bioinformatics **2006**; 22(21): 2688-90.

15. FigTree: Molecular evolution, hylogenetics and epidemiology. Available at: <http://tree.bio.ed.ac.uk/software/figtree/>.

16. Inouye M, Dashnow H, Raven L-A, et al. SRST2: rapid genomic surveillance for public health and hospital microbiology labs. Genome medicine **2014**; 6(11): 90.

17. Gupta SK, Padmanabhan BR, Diene SM, et al. ARG-ANNOT, a new bioinformatic tool to discover antibiotic resistance genes in bacterial genomes. Antimicrob Agents Ch **2014**; 58(1): 212-20.

18. Carattoli A, Zankari E, García-Fernández A, et al. In silico detection and typing of plasmids using PlasmidFinder and plasmid multilocus sequence typing. Antimicrob Agents Ch **2014**; 58(7): 3895-903.
